# Liquid Biopsy in Pre-Metastatic Niche: From Molecular Mechanism to Clinical Application

**DOI:** 10.3389/fimmu.2022.958360

**Published:** 2022-07-15

**Authors:** Zaoqu Liu, Ying Kong, Qin Dang, Siyuan Weng, Youyang Zheng, Yuqing Ren, Jinxiang Lv, Na Li, Yilin Han, Xinwei Han

**Affiliations:** ^1^ Department of Interventional Radiology, The First Affiliated Hospital of Zhengzhou University, Zhengzhou, China; ^2^ Interventional Institute of Zhengzhou University, Zhengzhou, China; ^3^ Interventional Treatment and Clinical Research Center of Henan Province, Zhengzhou, China; ^4^ Department of Colorectal Surgery, The First Affiliated Hospital of Zhengzhou University, Zhengzhou, China; ^5^ Department of Cardiology, The First Affiliated Hospital of Zhengzhou University, Zhengzhou, China; ^6^ Department of Respiratory and Critical Care Medicine, The First Affiliated Hospital of Zhengzhou University, Zhengzhou, China

**Keywords:** pre-metastatic niche, liquid biopsy, tumor microenvironment, molecular mechanism, clinical application

## Abstract

Metastatic dissemination represents a hallmark of cancer that is responsible for the high mortality rate. Recently, emerging evidence demonstrates a time-series event—pre-metastatic niche (PMN) has a profound impact on cancer metastasis. Exosomes, cell-free DNA (cfDNA), circulating tumor cells (CTC), and tumor microenvironment components, as critical components in PMN establishment, could be monitored by liquid biopsy. Intensive studies based on the molecular profile of liquid biopsy have made it a viable alternative to tissue biopsy. Meanwhile, the complex molecular mechanism and intercellular interaction are great challenges for applying liquid biopsy in clinical practice. This article reviews the cellular and molecular components involved in the establishment of the PMN and the promotion of metastasis, as well as the mechanisms of their interactions. Better knowledge of the characteristics of the PMN may facilitate the application of liquid biopsy for clinical diagnosis, prognosis, and treatment.

## Introduction

**Figure 1:**
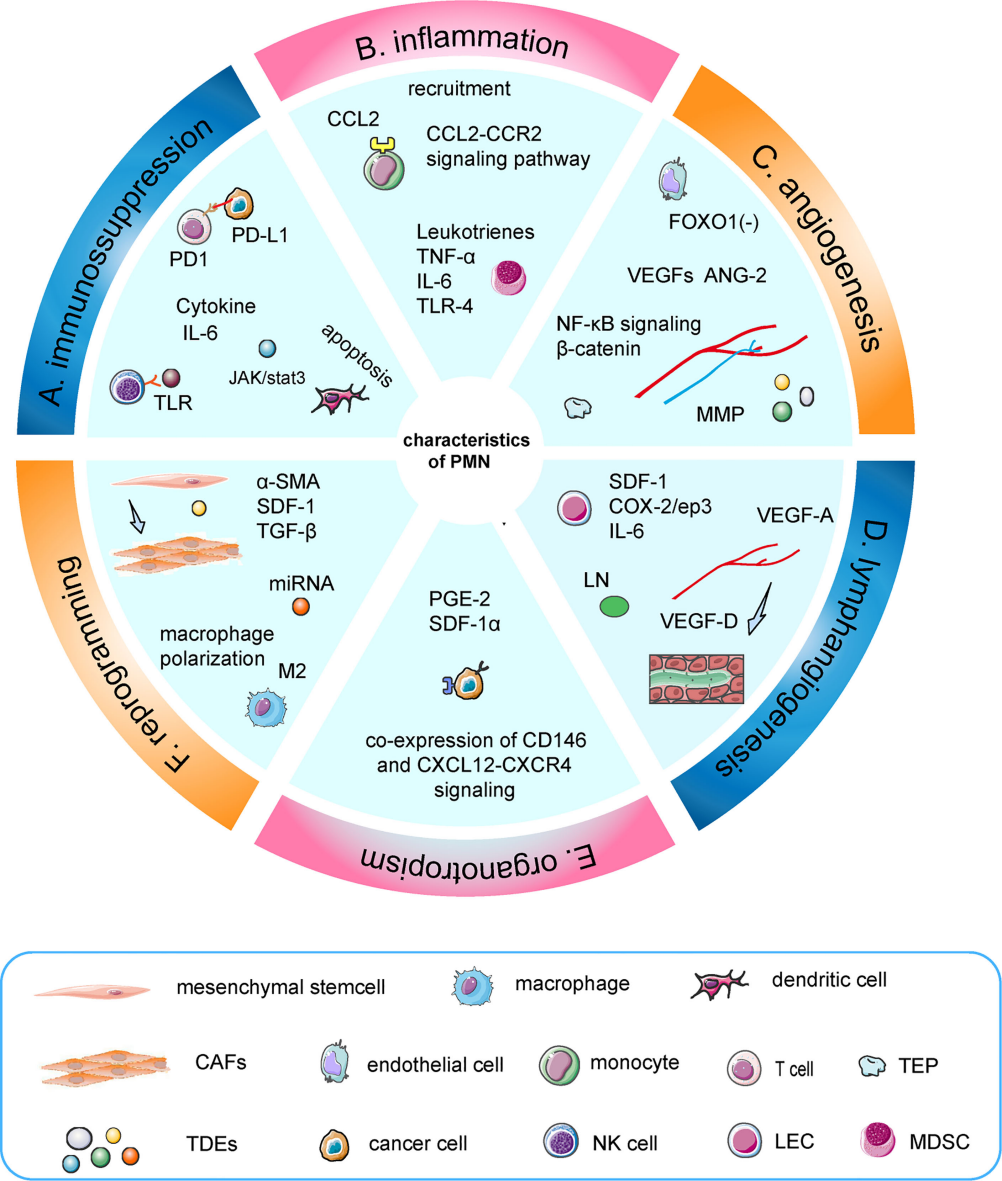
Characteristics of the PMN. **(A)** TDEs induced apoptosis of dendritic cells, increased secretion of IL-6, and inhibited the function of T cells and NK cells. **(B)** TNF-α induced S100A8-SAA3-TLR4 signaling and maintained an inflammatory state, which was mediated by MDSC. Similarly, IL-6, leukotrienes, and CCL2–CCR2 signaling pathways were involved. **(C)** Upregulation of angiopoietin-2 and VEGFs could regulate the angiogenic switch, in which TEPs and MMPs had a synergistic effect. Moreover, miR-135b promotes angiogenesis by inhibiting FOXO1 expression in endothelial cells. **(D)** VEGF-A and VEGF-D are crucial factors in the induction of premetastatic lymphangiogenesis. Furthermore, dendritic cells induce a PMN during LN metastasis through COX-2/ep3-dependent induction of SDF-1. **(E)** Tumor cells interacted with resident mesenchymal stem cells/pericytes around the surrounding blood vessels to achieve organotropism through co-expressed CD146 and Sdf-1/CXCL12-CXCR4 signaling. **(F)** Exosomes induced the transformation of mesenchymal stem cells into CAFs and macrophage M2-like polarization.

Distant metastasis was the terminal stage of tumor progression and the crucial cause of tumor death (1, 2). The immaturity of early diagnostic techniques and drug resistance indirectly promoted distant metastasis, leading to a high mortality rate of cancer (3, 4). Cancer progression is a dynamic process. Metastasis is an organ-selective and multi-stepping complex process that requires in-depth study, in order to find a better approach to diagnosis and treatment (5). The early perspective was that tumor cells migrated out of the primary site into the lymphatics or the bloodstream, survived in the circulation, and extravasated into the tissue, eventually forming metastasis (6). However, this theory was not enough to guide tumor-specific diagnosis and treatment. Stephen et al. first proposed the concept of “seed and soil” theory, which emphasized the importance of the microenvironment and revealed the organotropism of metastasis. The secondary site has established an abnormal, tumor growth-favoring microenvironment before tumor cells arrive (7). These predetermined soil microenvironments were termed “pre-metastatic niches”, which actively attracted the colonization of tumor cells (8). With the characteristics of inflammation, immunosuppression, angiogenesis/vascular permeability, reprogramming, organotropism, and lymphangiogenesis, the indispensable role of the PMN in metastasis has attracted increasing attention in recent years (9–13) (**Figure 1**).

Nowadays, tissue biopsy, as the gold standard of cancer identification, remains the first-line clinical mean (14). However, conventional tissue biopsies are invasive and sometimes only small samples can be obtained (15), making it impossible to characterize tumor heterogeneity or dynamically monitor tumor progression (16, 17). Moreover, it is restricted by tissue excision site, adverse accuracy and sensitivity, and high procedural expenses (18–20). Therefore, a novel diagnostic method has emerged—liquid biopsy. It is a neoteric skill to identify tumor markers among the accessible samples, such as cfDNA or RNA, circulating tumor DNA (ctDNA), CTCs, exosomes, circulating tumor-derived endothelial cells (CTECs), tumor-educated blood platelets (TEPs), and protein molecules (21–25). Biomarkers for liquid biopsies can be derived from cerebrospinal fluid (CSF), saliva, blood, ascites, urine, stool, and pleural fluid (26–28). Better than tissue biopsy, liquid biopsy is non-invasive, easier to repeat, and could better overcome tumor heterogeneity due to the wide range of samples (16, 29). In accord with the patient’s will not only from the macro perspective but also from the micro mechanism analysis, the progress of gene detection technology supports the development of the clinical application. Whole-exome sequencing or whole-genome sequencing data analysis explores the changes of genes and tumor burden in the course of patients (30), carries out comprehensive dynamic monitoring at the molecular level in a non-invasive manner, predicts tumor progression, and provides support for the formulation of subsequent precise treatment programs (31). Fortunately, cellular and molecular components such as exosomes, CTCs, and TEPs, which promote the formation of the PMN, are also widely present in plasma, urine, ascites, and other body fluids, making the application of liquid biopsy feasible (32, 33).

In this review, we focus on the critical molecular and cellular components that could be used in liquid biopsy at various stages of tumor metastasis niche formation and explain their clinical applications in prediction, prognosis, and treatment.

## The Evolution of PMN and the Process of Promoting Metastasis

**Figure 2:**
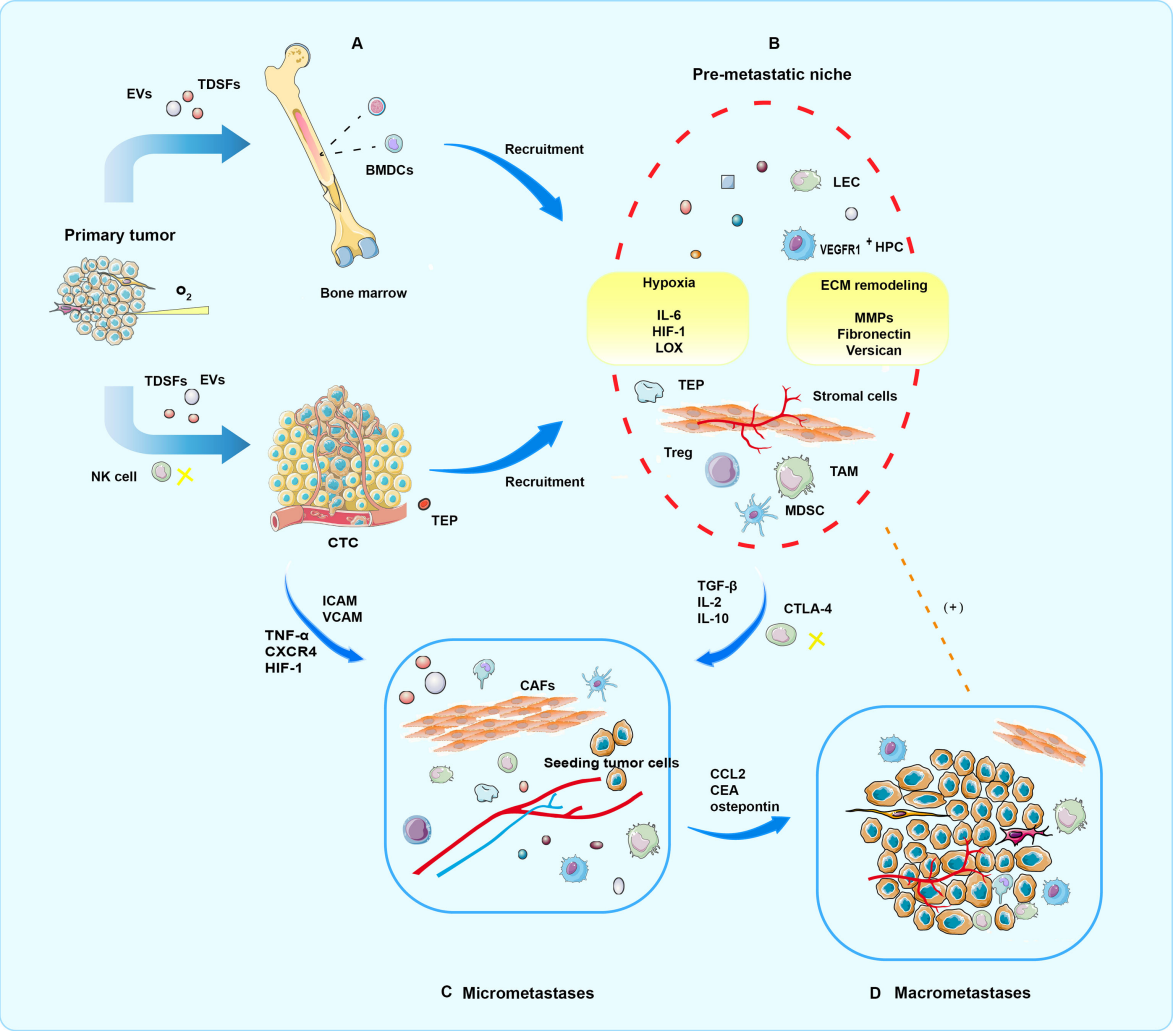
Four periods of PMN formation. **(A)** In the priming phase, produced by primary tumor cells, TDSFs, EVs, and other molecular components trigger an immature PMN formation in the secondary organ site or the same organ outside the primary tumor. **(B)** In the licensing phase, BMDC, regulatory, and suppressive immune cells involved in ECM remodeling are mobilized and recruited into the secondary sites. Finally, a mature PMN prepared well for potential seeding and colonization of CTCs. In addition, hypoxia is a critical condition for progress. **(C)** In the initiation phase, partial CTCs arrive and survive at the fertile PMN, where tumor cell seeding, colonization, and outgrowth occur, eventually resulting in micrometastases. **(D)** In the progression phase, PMNs attract more tumor cells to colonize. Moreover, mutual promotion of tumor cells and PMN is locked in a vicious circle, which culminates in macrometastases.

PMNs evolve in phased, sequential, and distinct ways, with each stage contributing to the metastasis process in its way (8, 9). Complex molecular and cellular changes have taken place in PMN to support the growth of metastatic tumors in the future (34, 35). Some molecules or cells could serve as markers of liquid biopsy. To simplify the complex development of time series events and more clearly explain the mechanism of liquid biopsy, the formation and metastasis promotion process of PMN can be divided into the following four temporal phases in sequence (9) (**Figure 2**).

### Priming

During the priming period, when the primary tumor proliferates uncontrollably, hypoxia and inflammation are produced, which induces the secretion of extracellular vesicles (EVs), tumor-derived soluble factors (TDSFs), and other molecular components (36). Exosomes secreted by cancer cells under hypoxic conditions may remodel distant PMN. Exosomes were isolated from human prostate cancer cells under normoxic and hypoxic conditions, and their effects on key biomarkers associated with PMN in nude mice were observed. It was found that the exosomes produced under hypoxic conditions increased the levels of matrix metalloproteinases (MMP) and extracellular matrix proteins (fibronectin and collagen) as well as enhanced the number of CD11b+ cells at selective PMN sites (37). In addition, abnormal glycolysis and high lactate production may reduce tissue pH, which has been verified in the tumor mouse model. The average pH of the tumor stroma was prominently lower than the surrounding tissue.

Moreover, acidifying the tumor microenvironment is not conducive to roving immune cells, establishing an anergic state in human tumor-specific CD8+ T lymphocytes, and facilitating immune escape (38, 39). Acidic conditions also alter key phenotypes of malignancies and determine the type and quantity of exosomes released from tumor cells. Likewise, in prostate cancer, the microenvironment acidity exerts selective pressure to induce an upregulation of nanovesicle production, expressing both the exosome biomarker CD81 and prostate-specific antigen (PSA). The ratio of PSA-expressing exosomes in the plasma of cancer patients is significantly higher compared to benign prostatic hyperplasia and healthy individuals (40). Therefore, as a tool for early diagnosis and screening, PSA cancer exosomes are considered novel and non-invasive.

Pervasively, TDSFs would serve as tumor precursors for organ-specific preparation of PMNs prior to CTC colonization. TDSFs, mainly including cytokines, chemokines, and growth factors, can contribute to the recruitment and activation of inflammatory cells (41). Many types of cancer are characterized by aberrant IL-6/JAK/STAT3 activation, which strongly inhibits the antitumor immune response (42). Besides IL-6, TNF-α and TGF-β also affect the myeloid-derived suppressor cell (MDSC) recruitment in the premetastatic lung, which is modulated by the generation of inflammatory chemoattractants S100A9 and S100A8 (43). In breast cancer, with CCR2 (the chemokine receptor for CCL2) expressed, inflammatory monocytes are more promising to accumulate in the pulmonary metastases than in primary tumors. Metastatic seeding is also suppressed by inhibition of the CCL2–CCR2 pathway and exhaustion of tumor-derived CCL2. All these suggest that a CCL2 overexpression improves macrophage infiltration and worsens the prognosis of human breast cancer (44). Moreover, CXCR2-positive MDSCs are attracted to metastatic sites by CXCL1 derived from tumor-associated macrophages (TAM) due to the stimulation of VEGFA released from primary tumor cells (45). From these results, it can be seen that hypoxia and acidic conditions are two key factors that lay the foundation for remodeling the matrix microenvironment, while also regulating tumor progression, recruitment, and activation of inflammatory cells, as well as facilitating the production of bioactive molecules.

### Licensing

In the licensing phase, under the action of TDSFs and EVs, a continuous flow of regulatory/suppressive immune cells and bone marrow-derived cells (BMDCs) is activated and mobilized into the transfer sites, which start to re-educate the stromal environment of the distant secondary sites to form a soil environment suitable for CTC colonization. Exosomes play a profound role in this stage (46). Extracellular vesicles, messengers between primary tumor sites and metastatic sites, are indispensable in forming PMN. Studies have shown that extracellular vesicles are small membrane vesicles from cells during activation and apoptosis. They can be divided into at least three types: apoptotic bodies, microvesicles, and exosomes with a size ranging from 40 to 100 nm (47, 48).

Interestingly, the biological functions of exosomes are different according to their sources (49). Tumor-derived exosomes (TDEs) carry a variety of bioactive components (nucleic acid, protein, and lipid), which are effective tools for intercellular communication. Exosomal microRNAs are the major messengers to execute these functions among these bioactive components. For instance, microRNA can establish an immunosuppressive environment by changing the phenotype and function of various immune cells. It is reported that MDSCs mediate the immunosuppressive environment, and hypoxia promotes the secretion of TDEs. MDSCs ingest TDEs, and tumor exogenous miR-9 and miR-181a targeted SOCS3 and PIAS3 to activate the JAK/STAT signal pathway, thus affecting the differentiation and activation of MDSCs and enhancing its immunosuppressive effect (50, 51). TDEs could not only directly inhibit NK cells’ toxicity and T cells’ proliferation but also achieve immunosuppression by enhancing the immune tolerance mediated by regulatory T cells. In terms of matrix remodeling, TDEs trigger the differentiation of mesenchymal stem cells (MSCs) to carcinoma-associated fibroblasts (CAFs) *via* the TGF-β/Smad pathway (52, 53). CAFs can modify the surrounding ECM to generate an ecological niche supporting cancer cell invasion, as well as facilitate cancer cell migration and invasion so as to promote tumor occurrence, metastasis, and diffusion to malignancy (35).

Furthermore, hypoxia stimulates hypersecretion of TDEs, enhances the recruitment of TDE to macrophages, and promotes M2-like polarization. In gastric cancer, miR-135b accelerates angiogenesis due to the inhibition of FOXO1 expression from endothelial cells (54). Notably, the critical role of exosomal proteins in promoting the production of PMN and as markers of liquid biopsy should not be ignored. For example, exosomes containing metalloproteinases promote angiogenesis, exosomal PD-L1 enhances immunosuppression, and exosome-induced T-cell inhibition depends on ICAM-1-mediated adhesion (55–57). From the above description, it could be demonstrated that exosomes are reasonable and practical as liquid biopsy biomarkers.

During this period, lymphangiogenesis is critical in the PMN, in addition to immunosuppression, angiogenesis, and matrix remodeling. Lymph node (LN) metastasis is a pivotal prognostic indicator of disease stage; thereby, the lymphatic system is considered to be a vital route of metastatic spread except for hematogenous metastasis (58). Partial tumors secrete lymphatic growth factors, which induce lymphangiogenesis and act on lymphatic vessels to boost metastasis. Namely, in the interior of the primary tumor or metastatic sites, such as sentinel LN (59), the original blood vessels form new lymphatic vessels and participate in regulating the immune response to the tumor under the action of TDSFs (60, 61). Clinical evidence indicates that tumor-derived VEGF-D and VEGF-A are key factors in the induction of premetastatic lymphangiogenesis in sentinel LN.

Moreover, since VEGF-D is associated with higher LN metastasis, it may be a potential predictor of positive LN metastasis in patients (62). Furthermore, when LN metastasis occurs, dendritic cells induce a PMN through COX-2/ep3-dependent SDF-1, suggesting that restraining this signaling axis may be an available measure to inhibit PMN formation and LN metastasis (63). Crucially, immunosuppression is facilitated by the presence of lymphatic vessels in the PMN, through which exosomes can reach mouse and human melanoma. In LN, exosomes preferentially bind CD169+ macrophages, destroy them to escape immune recognition, invade the LN cortex, and disrupt humoral immunity *via* interacting with B cells (64). Lymphatic endothelial cells (LECs), a composition of lymphatic vessels in the PMN, are regulated by TDSFs. Mechanistically, IL-6 induces the expression of HIF-1α, CCL5, and VEGF *via* the phosphorylation pathway to facilitate the recruitment, extravasation, and colonization of CCR5+ tumor cells in the niche, which lays the foundation for the smooth progress of the next stage.

Additionally, there is a self-reinforcing paracrine loop between cancer cells and LECs (65). Stromal lymphatic vessels are the primary route of metastasis in some cancers (66); moreover, the presence of LN metastasis worsens the prognosis (67). Consequently, an in-depth study on the molecular and cellular matrix and identifying critical factors in lymphangiogenesis may contribute to discovering new targets, which could be used in liquid biopsy to decrease tumor dissemination and improve prognosis.

### Initiation

In the initiation phase, through epithelial–mesenchymal transformation (EMT) (68, 69), tumor cells become CTCs and infiltrate from the blood vessels of the primary site. Some of them arrive and survive at the fertile PMN, where sowing, colonization, and proliferation of tumor cells occur, eventually leading to micrometastases. Nevertheless, before the arrival of CTCs, BMDCs migrated to distant sites induced by factors of the primary tumor, such as TNF-α, TGF-β, and TDEs. BMDCs contribute to tumor vascularization and neoplastic cell migration, which are latent promoters of CTC extravasation with organotropism (70).

Tumor cells migrating from the primary site to the PMN need to go through several critical stages: intravasation, intravascular survival, extravasation, and colonization of secondary sites. CTCs acquire enhanced migratory and invasive abilities through EMT (71), which is a transient, reversible process of cell differentiation. TAMs usually play a protumoral role, providing conditions for PMN and promoting extravasation, survival, and the continuous growth of tumor cells (72). EMT, a vital sign of solid tumors, has recently been shown to be an essential driver of macrophage polarization (71). EMT-colorectal cancer (CRC) programmed cells not only stimulate the production of various cytokines, such as IL-4 and CCL2, but also deliver exosomes directly to macrophage activation signaling cascade targets that directly inhibit programmed cell death at the post-transcriptional level, thereby enhancing M2-like polarization (73–75).

TEPs are promoters and protectors of blood metastasis. Entanglements of platelets and fibrin surrounding tumor cells protect CTCs from NK lysis (76). This makes it possible for CTCs to survive within the vasculature (immune evasion) and spread from the bloodstream. Moreover, activated platelets may facilitate vessel growth and maintain vascular integrity during tumor development (77). In addition, TEPs enhance the adhesion between CTCs and vascular endothelial cells through a selectin-dependent pathway to prepare for CTC extravasation (78). The function of TEP, a biomarker trove for liquid biopsy, has been proved, especially RNA (79). Particle-swarm optimization (PSO)-enhanced algorithms diagnose cancer, exploiting selected gene panels from TEP, which has also been demonstrated for accuracy in early and advanced non-small cell lung cancer (NSCLC) diagnosis. Consequently, TEPs possess the potential value as a liquid biopsy for various clinical and investigational applications (22, 80).

Although the role of CTCs as tumor biomarkers of liquid biopsy for research and clinical diagnosis has been widely concerned (81–83), isolating CTCs is a technical challenge owing to the rarity and heterogeneity of CTCs. Nevertheless, microfluidic-based isolation technologies are expected to break this limitation and promote the transformation of cancer clinical diagnosis and treatment mode (84). Technological advances make it feasible to convert from CTC counting to the thorough analysis of the CTC gene panels, transcriptome, protein, epigenome, and various functional characteristics, which can be used to monitor prognosis, anticipate micrometastasis, and act as an auxiliary means of tumor staging (85).

### Progression

In the progression phase, micrometastases attract more tumor cells to colonize, directly or indirectly promoting further microenvironment maturation by producing cytokines. This enables metastatic cancer cells to grow, invade, and progress at the site, creating a vicious cycle that culminates in macrometastases. For example, in bone metastases, there are two types of tumors: osteoblastic (bone-forming) and osteolytic (bone-lysing), of which prostate bone metastases are often the former and breast cancer bone metastases are the latter (86, 87). Several studies have shown that in osteolytic bone metastases, bone-derived chemokines and growth factors as chemoattractants, such as monocyte chemoattractant protein 1 and stromal cell-derived factor 1, could attract tumor cells to bone. Likewise, the interaction between bone marrow stromal cells and tumor cells could lead to increased production of growth factors and cytokines, further promoting PMN formation and attracting tumor cell colonization. For instance, the ligand for receptor activator of nuclear factor kappaB or IL-6 could accelerate angiogenesis, bone destruction, and tumor growth (88). This vicious cycle between bone microenvironment and tumor cells leads to osteoclastic lesions evolving, which macroscopically manifests as malignant metastasis of the tumor, a fatal event with a poor prognosis. Therefore, there is a demand for detecting tumor cells or components of the PMN prior to macrometastases, which could be discovered by imaging, to block cancer metastasis before it is incurable.

## Clinical Application of Liquid Biopsy in PMN

The heterogeneity of cancer cells within tumors is an essential obstacle to curative effect. Current cancer treatments, such as surgery, radiotherapy, and chemotherapy drugs, often kill healthy cells and poison patients, which cannot overcome tumor heterogeneity well (89). Therefore, it is essential to understand the molecular basis of tumors, such as the formation of PMN and the emergence of new diagnostic techniques. The current trend is to use liquid biopsy technology to obtain cancer cells or cancer-related molecules, which can explore epigenetic changes and oncogene expression based on the molecular level and flexibly apply the relevant results to clinical diagnosis and treatment. The table lists some critical molecular and cellular components in the process of PMN formation that can be detected by liquid biopsy, as well as their clinical applications (**Table 1**).

**Table 1:** Clinical application of liquid biopsy.

	Clinical application	Reference
**Exosomes and their contents**		
MIF	Liver PMN formation and metastasis	(90)
Level of PD-L1	Patients with NSCLC are higher than normal people	(91)
miRNA-10b	Early diagnosis of PDAC	(92)
miR-200b, miR-200c, and miR-373	Poor outcomes in ovarian cancer	(93)
Circular RNAs	A novel potential diagnostic biomarker of CRC	(94)
Exosomes derived from M1-polarized macrophages	Immunopotentiators for a cancer vaccine	(95)
**Circulating cells and inflammatory related markers**		
The proportion of regulatory T cells	Identification of CRC patients versus healthy controls	(96)
EGF, macrophage-derived chemokine, IL-10, IL-6, and IL-8 levels	Predictive value in irinotecan/bevacizumab-based treatments	(97)
Quantification of Tregs and CD8+ T cells	Prognostic value	(98)
The number of EPCAM (+) CD44(+) CD47(+) MET (+) CTCs	Correlated with lower overall survival and increased number of metastatic sites	(99)
cfDNA TF profiling	Detection of early-stage colorectal carcinomas	(100)
**Tumor microenvironment-derived markers**		
TIMP-1 + metalloproteinases	Prediction of patients’ survival	(101)
MSCs	Therapeutic production of exosomes	(102)
Transcriptomic analysis of CECs	Differentiation between healthy controls and CRC early stages	(103)
Identification and quantification of CECs	Monitoring clinical response and outcome	(104, 105)
Matrix metalloproteinase	With diagnostic value	(106)
VEGF-A and ICAM-1 variant	Prognosis value in bevacizumab treated patients	(107)
VEGF, HGF, EGF, and PDGF-AA levels	Predictive value in chemotherapy-based treated patients	(108)

CECs, circulating endothelial cells; EGF, epidermal growth factor; MIF, migration inhibitory factor; TF, transcription factor.

### Diagnostic Value

Early detection by liquid biopsy for cancer is promising (109). In the early stages of the disease, CTCs are already circulating in the blood prior to clinical evidence of metastasis. 5-Hydroxymethylcytosine signatures in cfDNA are highly predictive for colorectal and gastric cancer as an ideal diagnostic biomarker for human cancers, which are superior to conventional biomarkers, and comparable to the 5-hydroxymethylcytosine biomarker in tissue biopsy (110). Additionally, as a liquid biopsy marker, the urine epigenetic biomarkers have manifested satisfactory sensitivity and specificity in detecting upper tract urinary carcinoma (111). The effect of extracellular vesicles and particles (EVPs) in tumor detection and determination of cancer type has been demonstrated. Analyzing the protein contents of EVPs distinguished tumors from nearby noncancerous tissue and profiling extracellular vesicle proteins obtained from plasma may also reveal cancer type.

Moreover, both tissue-derived and plasma-derived EVPs were detected with high specificity (112). As an emerging biomarker for early and minimal malignancy diagnosis, exosomal microRNA has captured people’s attention because of its stability in multiple body fluids (113). Serum miR-378 levels were analyzed in 60 normal controls and 103 NSCLC patients. In NSCLC patients, exosomal miR-378 was significantly overexpressed, and its upregulation was associated with advanced TNM stage and positive LN metastasis. Additionally, the combination of serum exosomal miR-378 expression and carcinoembryonic antigen (CEA) had a high discriminating power to differentiate NSCLC subjects from controls (114). Similarly, the role of exosome-encapsulated microRNAs as a circulating diagnostic marker for low alpha-fetoprotein hepatocellular carcinoma has been demonstrated (115).

### Prognostic Value

The prognostic role of CTC counts as a tool for liquid biopsy can be seen in a variety of cancers (116–118). Previous studies have reported a lack of identification of novel biomarkers associated with breast cancer (119). Research on estrogen receptor-positive breast cancer suggested that independent prognostic information required for late clinical recurrence could be obtained from a single positive CTC assay (120). The presence of CTC is related to adverse prognosis in patients with metastatic CRC. While the presence of CTC weeks after surgery is not noticeably associated with CRC-related survival (CCRS) and recurrence-free survival (RFS) for patients with non-metastatic CRC, the association increases remarkably with time. Similarly, the presence of CTC in patients with optimistic preoperative staging was connected with a significant reduction in RFS and CCRS (121). In a prospective trial, CSF-derived cfDNA copy number variations were used as a surrogate for minimal residual disease (MRD) to detect disease progression (122). These phenomena may demonstrate that highly sensitive liquid biopsy assays can be applied to detect and characterize MRD (123). Blood tests based on CTC phenotype simulations can also assess overall survival and tumor metastasis in pancreatic ductal adenocarcinomas (PDAC) patients. CTC transcriptional profiling can be used not only as an independent prognostic marker but also to determine the emergence of multiple androgen receptor signaling inhibitors resistance mechanisms, which can guide the choice of treatment options (83). Besides the prognostic assessment, liquid biopsy technology can achieve individualized management of clinical patients. For instance, in stage II colon cancer, ctDNA-guided therapy reduces adjuvant chemotherapy use without compromising recurrence-free survival (124).

### Therapeutic Application

The clinical utility of CTC as a marker of liquid biopsy for prognosis and monitoring of systemic treatment response was reported a decade ago (125). In recent years, the study of ctDNA, circulating cfRNA, EVs, and TEPs has also attracted much attention (126–128). The serum level of miR-378 in 73 patients with NSCLC decreased significantly after radiotherapy, which can be used as an indicator of the efficacy of radiotherapy for NSCLC (114). The effect of immunotherapy on cancer patients can be evaluated by liquid biopsy. In one study, patients who tested positive for ctDNA showed improvement in disease-free survival and overall survival when receiving adjuvant atezolizumab instead of observation (129).

Additionally, it was attractive that exosomal miRNAs conveyed the drug resistance message. Exosomes miR-3913-5p and miR-184, as biomarkers of osimertinib resistance, are suitable for NSCLC patients to detect their expression. This may be related to the abnormal activation of alternative pathways (PI3K pathway activation and RAS-MAPK pathway abnormality), indicating that miRNAs derived from peripheral blood exosomes are involved in the resistance mechanism of osimertinib through the pathway. In addition, miR-433 can inhibit cisplatin chemoresistance by regulating DNA damage and inactivating the WNT/β-catenin signaling pathway by targeting p24 transporter 5 in NSCLC. These studies suggest that miRNA can provide latent therapeutic targets for patients with NSCLC (130). The homing effect of exosomes on primary tumor cells is promising for targeted therapy. For example, PMN mimics, engineered biomaterials embedded with ovarian cancer exosomes into the peritoneal cavity of mouse models, can effectively recruit and capture free ovarian tumor cells in ascites, thereby arresting colonization in normal pelvic organs, reducing metastasis, and improving patient survival (131, 132). Furthermore, exosomes can also be used as a delivery system to load drugs and improve drug spillover in tumors (133). Generally, therapeutic applications, such as therapeutic response monitoring, targeted therapy, and drug resistance detection, have enriched remedies for cancer and are expected to enhance efficacy.

## Opportunities and Challenges of Liquid Biopsy Technology in Clinical Practice

Compared with tissue biopsy and imaging diagnosis, liquid biopsy possesses the advantage of being non-invasive, repeatable, and economical, and having an early diagnosis, which could surmount the temporal and spatial heterogeneity of tumors (134). With the improvement of liquid biopsy technology, its sensitivity and operability have also been significantly strengthened. For example, CAPP-seq personalized cancer analysis technology is an economical and susceptible method to quantify ctDNA. In NSCLC, ctDNA levels are closely associated with tumor volume, distinguishing between treatment-related imaging transformations and residual disease, allowing for earlier response assessment and personalized cancer treatment (135). There are also many other detection techniques such as polymerase chain reaction-based, microfluidic methods, chip-based, next-generation sequencing-based, and fluorescence *in situ* hybridization-based (136). However, the specificity of liquid biopsy results poses some challenges. Firstly, gene mutations associated with cancer occur with age, even in people who have never experienced cancer. Therefore, while technological advances have made ctDNA testing more specific, false-positive results from its use in cancer screening can cause significant anxiety (137, 138). Secondly, widespread clinical application of liquid biopsy technology remains unrealistic because the standardization and replication of test results are challenged (139). However, advances in the characterization and detection of ctDNA and the application of single-gene and multi-gene detection methods have made the clinical application of targeted therapy possible. In addition, the application of liquid biopsy in the systemic treatment of sufferers with “ctDNA relapse” has also been noted. This is a new concept to detect cancer recurrence by detecting ctDNA after treatment, which is earlier than imaging examination (140). The advanced technology currently used for liquid biopsy is the detection of exosomes derived from cancer with biosensors, with highly specific target selection (125). Single-cell sequencing has increased the understanding of the molecular pathways involved in triggering cancer progression (141). Molecular imaging, especially when combined with liquid biopsy for screening, promises early disease localization because biochemical changes precede anatomical changes (142). The methylation patterns informed by cfDNA sequencing can be used for epigenetic variation assessment, with potential value for early detection of fatal malignancies (143). In conclusion, the emergence and development of these new technologies have contributed to the evolution of precision medicine.

## Conclusion and Perspectives

As a non-invasive, reproducible method, liquid biopsy has achieved remarkable success in the early detection and tracking of biomarkers. Furthermore, biological interactions between the tumor microenvironment and PMN are increasingly vital as potential mechanisms of tumor progression. In this process, the role of soluble factors, exosomes, and circulating cells from the tumor microenvironment has been emphasized as neoplastic markers for cancer diagnosis, prediction and prognosis, therapeutic response monitoring, and therapy guidance. However, the liquid biopsy technique demands a breakthrough in clinical practice. For example, the high heterogeneity and nanoscale size of exosomes pose great technical difficulties for the isolation and detection of their molecular information. There are various methods of detecting CTCs and ctDNA, and the diagnostic procedures are not standardized, which requires high enrichment technology of CTC in blood. These problems need to be overcome to achieve widespread clinical application. Early diagnosis and blocking of cancer progression before the formation of micrometastases or even PMN is a promising research direction, which requires liquid biopsy technology to break through the limitations and be flexible for clinical practice. In summary, as the three branches of liquid biopsy, CTC, ctDNA, and exosomes are crucial components for the preparation and biological function of PMN. Therefore, liquid biopsy diagnosis clarifies the biological characteristics of PMN, possibly changing the process at the initial stage of metastasis, and has tremendous potential in precision medicine.

## Author Contributions

ZL, XH, and QD provided direction and guidance throughout the preparation of this manuscript. YK, QD, and ZL wrote and edited the manuscript. QD, JL, NL, and YH reviewed and made significant revisions to the manuscript. SW, YZ, YR, and ZL collected and prepared the related papers. All authors read and approved the final manuscript.

## Conflict of Interest

The authors declare that the research was conducted in the absence of any commercial or financial relationships that could be construed as a potential conflict of interest.

## Publisher’s Note

All claims expressed in this article are solely those of the authors and do not necessarily represent those of their affiliated organizations, or those of the publisher, the editors and the reviewers. Any product that may be evaluated in this article, or claim that may be made by its manufacturer, is not guaranteed or endorsed by the publisher.

## Abbreviations

BMDCs, bone marrow-derived cells; CAFs, carcinoma-associated fibroblasts; CCRS, CRC related survival; CEA, carcinoembryonic antigen; CECs, circulating endothelial cells; cfDNA, circulating cell-free DNA; CRC, colorectal cancer; CSF, cerebrospinal fluid; CTCs, circulating tumor cells; ctDNA, circulating tumor DNA; CTECs, circulating tumor-derived endothelial cells; EGF, epidermal growth factor; EMT, epithelial–mesenchymal transformation; EVs, extracellular vesicles; EVPs, extracellular vesicles and particles; LECs, lymphatic endothelial cells; LN, lymph node; MDSCs, myeloid-derived suppressor cells; MIF, migration inhibitory factor; MMP, matrix metalloproteinases; MRD, minimal residual disease; MSCs, mesenchymal stem cells; NSCLC, non-small cell lung cancer; PDACs, pancreatic ductal adenocarcinomas; PMNs, pre-metastatic niches; PSA, prostate-specific antigen; PSO, particle-swarm optimization; RFS, recurrence-free survival; TAM, tumor-associated macrophages; TDEs, tumor-derived exosomes; TDSFs, tumor-derived soluble factors; EPs, tumor-educated blood platelets; TF, transcription factor.
